# The role of protein acetylation in carcinogenesis and targeted drug discovery

**DOI:** 10.3389/fendo.2022.972312

**Published:** 2022-09-12

**Authors:** Jingru Yang, Cong Song, Xianquan Zhan

**Affiliations:** ^1^ Shandong Key Laboratory of Radiation Oncology, Shandong Cancer Hospital and Institute, Shandong First Medical University and Shandong Academy of Medical Sciences, Jinan, China; ^2^ Medical Science and Technology Innovation Center, Shandong First Medical University, Jinan, China

**Keywords:** acetylation, HAT, HDAC, post-translational modification, cancer, HDAC inhibitor

## Abstract

Protein acetylation is a reversible post-translational modification, and is involved in many biological processes in cells, such as transcriptional regulation, DNA damage repair, and energy metabolism, which is an important molecular event and is associated with a wide range of diseases such as cancers. Protein acetylation is dynamically regulated by histone acetyltransferases (HATs) and histone deacetylases (HDACs) in homeostasis. The abnormal acetylation level might lead to the occurrence and deterioration of a cancer, and is closely related to various pathophysiological characteristics of a cancer, such as malignant phenotypes, and promotes cancer cells to adapt to tumor microenvironment. Therapeutic modalities targeting protein acetylation are a potential therapeutic strategy. This article discussed the roles of protein acetylation in tumor pathology and therapeutic drugs targeting protein acetylation, which offers the contributions of protein acetylation in clarification of carcinogenesis, and discovery of therapeutic drugs for cancers, and lays the foundation for precision medicine in oncology.

## Introduction

Cancer is a malignant disease with heterogeneity, and its occurrence and development are affected by a variety of factors (1). It has strong ability to migrate, proliferate, and invade, and can adhere to the surrounding normal tissues. There are many factors to affect cancers, including genetic, epigenetic, and environmental factors, which all enhance tumor malignancy (2).

Epigenetics is the change in the level of gene expression without changes in the gene sequence (3). Abnormal changes in epigenetics may lead to the occurrence and development of various malignant diseases. Epigenetic research mainly includes DNA covalent modifications such as DNA methylation and poly-methylation, chromatin remodeling, and the regulation of gene expression levels by non-coding RNAs (3). Proteins are the ultimate executors of biological functions. Studies have shown that many abnormal post-translational modifications are closely associated with malignant tumors, such as acetylation, ubiquitination, and phosphorylation (4). Of them, protein acetylation was discovered in the 1960s, but acetylation has not been extensively studied until recent years (5). Acetylation occurs on histones and non-histones, and most of the current research focuses on acetylation on histones (6). Histone is an octamer that constitutes the ribosome, consisting of four core histones (H3\H4\H2A\H2B), which combine with surrounding DNA fragments to form subunits of the ribosome, and the histone tail is easily translated by different post-translational modification to affect chromatin state and gene expression (6). Histones are prone to be acetylated (6). Studies have shown that protein acetylation is closely related to transcriptional regulation (7).

Acetylation modification is the process of covalently binding acyl-CoA compounds to specific amino acid sites of proteins under the action of acetyltransferase, generally binding to lysine residues (8). This process can also be reversed by deacetylases. This process is reversible and plays an important role in chromatin remodeling, gene expression, and regulation of protein function (9). Acetylation processes in different organelles are independent of each other. For example, acetyl-CoA in mitochondria and acetyl-CoA outside mitochondria are independent of each other (8). Acetylation in mitochondria plays an important role in biological processes such as the tricarboxylic acid cycle and fatty acid oxidation (9, 10). Moreover, protein acetylation is involved in the transcriptional regulation of genes, and some transcriptional co-activators have acetylase activity and some transcriptional co-repressors have deacetylase activity (7). Protein acetylation is associated with novel drug targets for a variety of diseases such as cancer (11). Thereby, it emphasizes the important scientific merits of protein acetylation in carcinogenesis and targeted drug discovery.

This article reviews (i) the component and process of protein acetylation system in cancers, including types of acetylation (N-acetylation, O-acetylation, and K-acetylation), regulators of acetylation (writers-acetyltransferases, erasers-deacetylases, acetyl coenzyme A, and readers), (ii) biological role of acetylation in cancer pathophysiology, including apoptosis, autophagy, cellular metabolism, cell cycle, proliferation, migration, and invasion, and (iii) acetylation system-based targeted drugs in cancer, including HAT inhibitors, HAT activators, HDAC inhibitors, and BET inhibitors. Also, we proposed the future perspectives about the roles of protein acetylation in carcinogenesis and targeted drug discovery. In this review, we focus on the classification of acetylation and its impact on pathophysiological processes in tumorigenesis. We link protein acetylation with epigenetic drugs for tumor treatment to promote the development of cancer precision medicine.

## The components and process of acetylation system in cancers

### Types of acetylation in cancers

Protein acetylation is the process of covalently binding acyl-CoA class A compound to protein-specific amino acid sites under the action of acetyltransferases. Vincent Allfrey and his colleagues discovered histone lysine acetylation modification in 1964 (7). In subsequent studies, they gradually discovered the mechanism of acetylation modification, the discovery and identification of HAT and HDAC, and the discovery and identification of reader domains, which laid the foundation for protein acetylation. With the development of mass spectrometry and proteomics, non-histone acetylation was discovered and the regulatory process of non-histone acetylation was revealed (7). More and more studies have proved that histone acetylation and non-histone acetylation have the same importance in the regulation of biological processes in organisms (7). After the discovery of non-histone acetylation, histone acetyltransferases were also renamed lysine acetyltransferases and histone deacetylases were renamed lysine deacetylases (7). Histone acetylation occurs in the nucleus and is a type of epigenetic regulation that regulates chromatin structure to regulate transcription and DNA repair. Histone hyperacetylation by histone acetyltransferase is associated with transcriptional activation, while histone deacetylation by histone deacetylase is associated with transcriptional repression. Histone acetylation promotes transcription by remodeling higher-level chromatin structure, attenuating histone-DNA interactions, and providing binding sites for transcription activation complexes (12). Histone deacetylation inhibits transcription, and histone deacetylation and acetylation maintain homeostasis by opposing mechanisms, including the assembly of higher-order chromatin structures and the exclusion of bromo domain-containing transcriptional activation complexes (12). Histone acetylation and tumorigenesis are also closely related, and histone acetylation promotes the expression of certain genes that can lead to tumors (13). For example, P300 is a histone lysine acetyltransferase that catalyzes the attachment of acetyl groups to lysine residues, which leads to the activation of several genes, including several oncogenes. Study finds elevated expression of p300 in breast cancer (13).

Non-histone acetylation is involved in most biological processes in organisms and occurs with very high frequency. Non-histone acetylation is involved in key cellular processes related to organism physiology and tumors, such as gene transcription, DNA damage repair, cell division, protein folding, autophagy, cell signaling, and metabolism. For example, HDAC6 acts not only on histones, but also on non-histone substrates to maintain the balance of non-histone acetylation (14). α-Tubulin, the first non-histone substrate of HDAC6, reversibly modulates its homeostasis and in turn affects MT stability and function (15). The α-tubulin acetylation affects intracellular trafficking events through the protein encoded by the cylindromatosis gene, thereby participating in mitosis and affecting the development of the cell cycle (14). Non-histone acetylation modifies protein expression through various mechanisms and affects protein function. For example, regulating protein stability, regulating protease activity, affecting subcellular localization, and regulating protein-protein interactions, etc. Protein acetylation can be classified into three types (N-acetylation, O-acetylation, and K-acetylation) according to acetylation site in a protein amino acid sequence.

#### *N*-acetylation

N-terminal acetylation in a protein is one of the most common modifications in mammals, which transfers the acetyl group to the N terminus of the protein, the amino group of the first residue in the protein (4). Unlike O-acetylation and K-acetylation, N-acetylation is an irreversible post-translational modification. N-acetylation occurs in 80%-90% of human proteins and is controlled by N-acetyltransferases. The addition of the acetyl group to N-terminus changes the charge carried by the amino acid, neutralizes the positive charge of the amino acid residue itself, changes the molecular weight of amino acid residue, changes the properties of the protein, and then affects the biological function of the protein. Studies have shown that N-acetylation mainly affects protein-membrane binding and protein stability (16). N-acetylation is also one of many factors contributing to tumor progression; for example, slow N-acetylation is a factor in bladder carcinogenesis and muscle invasiveness, and NAT1 is recognized as a biomarker candidate in bladder cancer and a potential target for drug development point (17).

#### O-acetylation

O-acetylation was detected less frequently than N-acetylation and K-acetylation. O-acetylation occurred mainly on the hydroxyl group at the serine or threonine terminal. O-acetylation was discovered in 2006 by Orth while studying YopJ, a bacterial virulence factor that acts as an acetyltransferase during acetylation (18). Studies have shown that YopJ transfers acetyl groups to the hydroxyl residues of serine or threonine, which inhibits the activation of MAPKK6, thereby inhibits the activation of MAPK and NF-κB pathways, inhibites the response of immune responses, and promotes the occurrence and development of malignant diseases (19). The discovery of O-acetylation adds to the complexity of the study of the regulation of gene expression by acetylation. Some studies have found that O-acetylation can compete with phosphorylation at some modification sites (20). Although there are few studies on O-acetylation, it has been found that O-acetylation is closely related to tumorigenesis in recent years (21). GD2 O-acetylation is elevated in neuroblastoma and glioblastoma, which is a potential biomarker of therapeutic target (21). In childhood acute lymphoblastic leukemia, the expression of 9-O-acetylated sialoglycoprotein was enhanced, decreased with the remission of clinical symptoms, and increased again when the disease relapsed (22). These studies indicate that O-acetylation might be a potential biomarker and target for drug-targeted therapy (22).

#### K-acetylation

Lysine acetylation is currently the most extensive research field of acetylation. Protein deacetylation is very extensive in the human body, with more than 3600 acetylation sites in more than 1750 proteins (23). Lysine acetylation mainly occurs on the histones of ribosomes and is jointly regulated by lysine acetyltransferase and lysine deacetylase to maintain the dynamic balance of lysine acetylation in cells (9). Lysine acetylation also occurs in non-histone proteins in the nucleus, cytoplasm, and mitochondria, and regulates various biological functions of cells (9). For example, DNA repair enzymes can be carried out in the nucleus through acetylation (24). The dynamic balance of lysine acetylation affects multiple functions in the cell, such as gene replication, gene transcription, stability of protein structure, interaction between proteins and proteins, cell cycle, cellular self-regulation, phagocytosis, and cell apoptosis (25). For example, there is a large amount of tubulin in the cytoplasm. Tubulin acts as a cytoskeletal component to maintain the stability of cells. The acetylation of α-tubulin is a significant marker of microtubule stability (26). Studies have shown that the acetylation of cytoskeleton is related to the occurrence of tumors, and tubulin is the target of many anti-tumor drugs. Lysine acetylation is one of the most important post-translational modifications in cell signaling pathways (10). The occurrence and development of many malignant tumors are closely related to lysine acetylation (27). For example, most metabolic enzymes are targets for lysine acetylation, such as ATM, ABL1, CDK9, BTK, CDK1 (25, 28–32), and a large number of acetylated proteins mediated abnormal changes in cell signaling pathways (33). For instance, acetylated phosphoglycerate kinase 1 is involved in glycolysis and amino acid biosynthesis in nonfunctional pituitary neuroendocrine tumors (NF-PitNETs) (34).

### Regulators of acetylation in cancers

**Figure 1:**
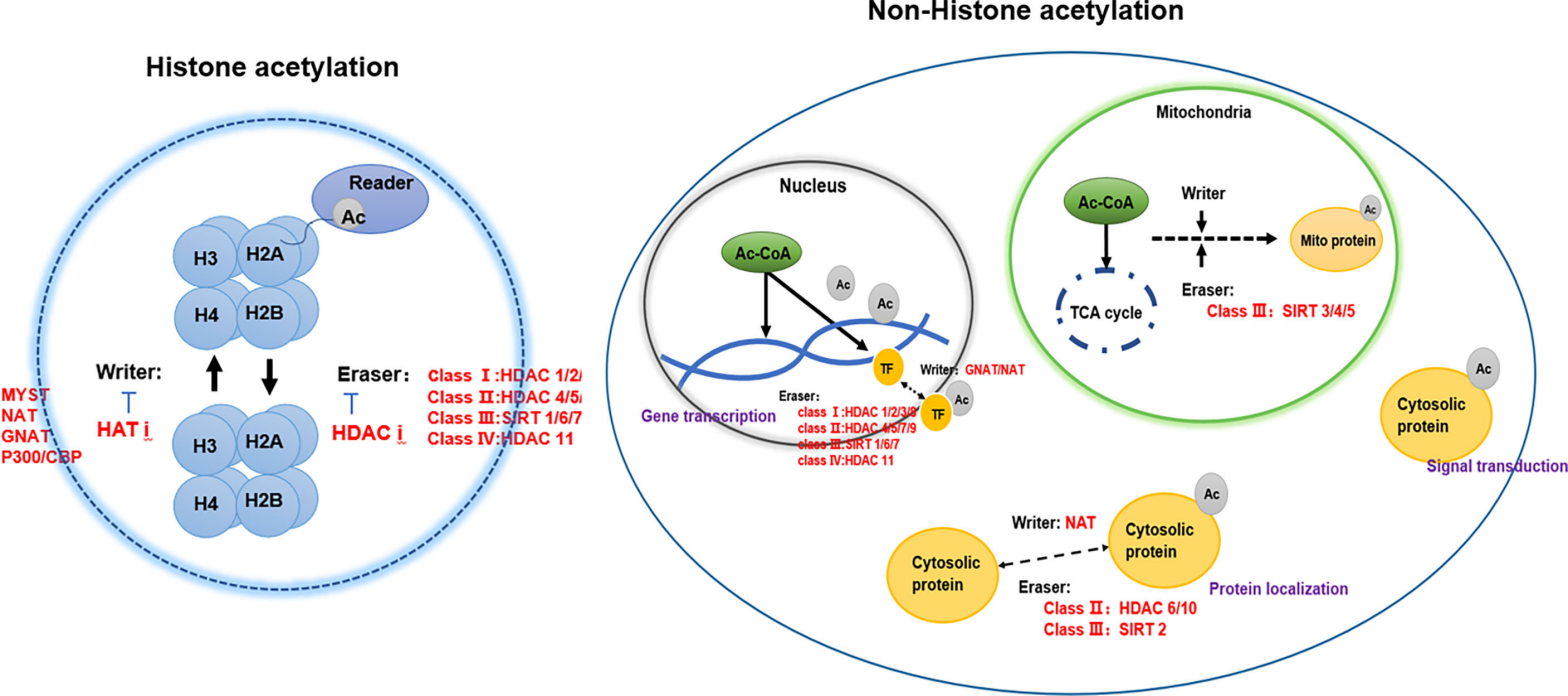
The regulators of protein acetylation-writers, erasers, and readers.

Acetylation in eukaryotic cells is in a dynamic equilibrium, which is jointly participated by writer-acetyltransferase, eraser-deacetylase, acetyl coenzyme A, and reader (4) (**Figure 1**).

#### Writer-acetyltransferases

Protein acetylation is a dynamic process by the joint action of acetyltransferases and deacetylases, including N-acetylation, O-acetylation, and K-acetylation (18). Most of the current studies focus on the acetylation of histones (6). Histone acetylation mainly occurs on lysine residues of histones (K-acetylation) in eukaryotic cells. The group is transferred to the side chain of the lysine residue, which in turn changes the R group of the lysine residue, neutralizes the positive charge on the lysine residue, and then affects the properties of the protein, and affects the structure and regulation of chromatin gene expression. According to structural and sequence similarity, mammalian lysine acetyltransferases are mainly divided into three categories: GCN5-related enzymes, p300-related enzymes, and MYST19-related enzymes (35). These acetyltransferases are present in the nucleus, and there are also acetyltransferases such as ESCO1, ESCO2, and HAT1 present in the nucleus (7). In addition to acetyltransferase in the nucleus, tubulin also contains acetyltransferase TAT1 (36). Acetylation of α-tubulin is a prominent marker of microtubule stability, and p27 promotes microtubule acetylation by binding and stabilizing ATAT-1 in glucose-deficient cells (37). Acetyltransferases have substrate specificity, which can regulate the structure of chromatin and thus regulate gene expression (7). For example, MOZ has a plant homeodomain-linked (PHD) type zinc finger that regulates chromatin by binding to trimethylated lysine 4 of histone 3 Structure (38). Acetyltransferases are also closely associated with transcriptional activators. For example, loss of Kat2a affects transcription factor binding and reduces transcriptional burst frequency in a subset of gene promoters, thereby enhancing variability at the transcriptional level (39). CBP/p300 blocks the role of estrogen receptor alpha (ERα) in luminal breast cancer by inhibiting enhancer H3K27 acetylation (40). The mechanism of action of acetyltransferase depends on oncogene activation, which is closely related to the occurrence and development of tumors through signal transduction (41). Both Tip60 expression and ABCE1 acetylation were up-regulated in lung cancer cells (42). Downregulation of Tip60 reduced ABCE1 acetylation levels and inhibited cell proliferation, invasion and migration (42). In addition, downregulation of Tip60 activates the apoptotic pathway, thereby achieves its inhibitory effect (42). Naa10 can acetylate and stabilize TSC2, thereby inhibiting mTOR activity and inhibiting cancer development (43). Acetyltransferase can also control the occurrence and development of tumors by regulating kinases in tumor cells. Naa10 inhibits tumor cell migration by inhibiting MYLK kinase activity through acetylation (44). ESCO2 inhibits the nuclear translocation of hnRNPA1 and increases the binding of hnRNPA1 (heterogeneous nuclear ribonucleoprotein A1) to the intron sequence flanking exon 9 (EI9) of PKM RNA, which ultimately inhibites the formation of PKM1 isoforms and induces the formation of PKM2 isoforms to promote glycolysis of tumor cells, and accelerate the metabolism of tumor cells (45).

The abnormal expression of HATs is usually associated with the occurrence and development of several malignant tumors and poor prognosis, which indicates that HATs may be potential tumor therapy targets and potential biomarkers (46). It is still necessary to in-depth study the effect mechanism of HATs on tumors to clarify the applicability and effectiveness of HATs in the clinical treatment of tumors (**Table 1**).

**Table 1:** Classification, localization and role of acetylases in cancers.

Family	Name	Location	Effects on cancers	Reference
NAT	Naa10 (NatA)	Nucleus	Represses tumor cell migration	(44)
	Naa20 (NAT5)			
	Naa30 (NAT12)	Cytoplasm		
	Naa40 (NAT11)		Negative regulator of apoptosis	(47)
	Naa50 (NAT5)			
	Naa60 (NAT15)			
	Naa11			
	NAT10			
GNAT	KAT1	Nucleus		
	GCN5 (KAT2A)		Deplete acute myeloid leukemia	(39)
	PCAF (KAT2B)		Modulate protein stability	(48)
	ELP3 (KAT9)		Wnt-driven intestinal tumor initiation	(49)
	ATAT-1			
	AT-1		Regulate apoptosis	(50)
	AT-2			
P300/CBP	CBP (KAT3A)		Breast cancer, hematological malignancies	(40, 51)
	P300 (KAT3B)		Breast cancer, hematological malignancies	(36, 52)
MYST	Tip60 (KAT5)		Lung cancer	(42)
	MOZ (KAT6A)		Acute myeloid leukemia	(38)
	MORF (KAT6B)		Leiomyoma	(53)
	HBO1 (KAT7)		Promotes DNA replication licening	(54)
	MOF (KAT8)		Tumor promoter in GBM	(55)
Others	ESCO1		Promote sister chromatid cohesion	(56)
	ESCO2		Promote LUAD malignant progression	(45)
	HAT1			
	TAT1	Tubulin		

#### Eraser-deacetylases

Protein deacetylase is called the “eraser” of acetyl group, which reduces the acetyl group attached to the amino acid residue to acetate, affects the R group structure of the amino acid residue, and then reduces the positive charge of the amino acid, and facilitates its binding to negatively charged DNA (52). Protein deacetylases are involved in regulating gene replication, gene transcription, protein structure stability, DNA damage repair, and other cellular functions (7). Mammalian genes encode 18 deacetylases, which act on histone and non-histone proteins in cells to remove their acetyl groups (7). For example, sirtuin enzymes are divided into four classes and localized in different locations of cells (57). Class III belongs to NAD+-dependent sirtuin enzymes, which are localized in mitochondria, cytoplasm and nucleus (57). Zn^2+^-dependent HDACs have a highly conserved deacetylase domain, including classes I (HDAC 1, 2, 3, and 8), II (HDAC 4, 5, 7, and 9), and IV (HDAC 11) localized in the nucleus (58). Studies have found that HDAC can not only act on histone deacetylation, but also play other roles on histones, such as decrotonylation, and desumoylation (9). HDACs have been found to be abnormally expressed or altered in localization in a variety of cancers (59). Studies have shown that the abnormal expression of HDAC in cancer patients is closely related to the dynamic imbalance of acetylation in the human body (60). In addition, the specific domains of individual sirtuins also have their own specific functions, such as maintaining protein stability (52) (**Table 2**).

**Table 2:** Classification, localization and role of deacetylases in cancers.

Family	Name	Location	Cancer	Effects on cancer	Reference
Class I	HDAC 1	Nucleus	Acute myeloid leukemia (AML) glioblastoma	Regulate apoptosis Maintenance of the malignant phenotype	(61–63)
	HDAC 2	Nucleus	Hepatocellular carcinoma (HCC)	Regulate cell cycle, migration, apoptosis, and cell adhesion.	(64, 65)
	HDAC 3	Nucleus	Acute myeloid leukemia (AML), colorectal cancer, lung cancer, melanoma, human maxillary cancer, acute promyelocytic leukemia (APL), multiple myeloma (MM), hepatocellular carcinoma (HCC), breast cancer	Promotes cancer progression	(66–72)
	HDAC 8	Nucleus	Acute myeloid leukemia	Aberrant expression or deregulated interactions with transcription factors	(73, 74)
Class II a	HDAC 4	Nucleus	Breast cancer, Glioblastoma nasopharyngeal carcinoma	Promote proliferation, migration, and invasion in nasopharyngeal carcinoma	(75–77)
	HDAC 5	Nucleus	CAN in HCC	Regulate cell proliferation and invasion, the immune response, and maintenance of stemness	(78, 79)
	HDAC 7	Nucleus	Breast cancer	Regulates gene expression, cell proliferation, cell differentiation and cell survival	(80, 81)
	HDAC 9	Nucleus	Breast cancers	Antiestrogen resistance, promotes tissue-specific transcriptional regulation	(82, 83)
Class II b	HDAC 6	Cytoplasm	Prostate cancer	Regulate cell proliferation, metastasis, invasion, and mitosis	(14, 84)
	HDAC 10	Cytoplasm	Lung adenocarcinoma		(85)
Class III	SIRT 1	Nucleus	Lung cancer	Involved in gene regulation, genome stability maintenance, apoptosis, autophagy, senescence, proliferation, aging, and tumorigenesis	(86, 87)
	SIRT 2	Cytoplasm	Lung cancer, Glioblastoma melanoma	suppresses NK cell function and proliferation	(76, 86, 88)
	SIRT 3	Mitochondria	Lung cancer, Ovarian cancer	Regulate autophagy	(89, 90)
	SIRT 4		NSCLC, Endometrioid adenocarcinoma		(91) (92)
	SIRT 5		Acute Myeloid Leukemia		(93)
	SIRT 6	Nucleus	Acute Myeloid Leukemia		(94)
	SIRT 7	Nucleosome	Breast cancer, glioblastoma		(76, 95)
Class IV	HDAC11	Nucleus	HCC	High expression in HCC	(96)

Transcription factors are a kind of non-histone proteins, and protein deacetylases regulate gene transcription activity by deacetylating transcription factors (27). For example, HDAC7 regulates the acetylation of H3K27 and the transcriptional activity of super-enhancer-related genes in breast cancer stem cells (80). A common mutation in AML is a chromosome 16 inversion that fuses the core-binding factor beta (CBFB) gene with the smooth muscle myosin heavy chain gene (MYH11) to form the oncogene CBFB-MYH11 (61). The expressed protein CBFbeta-SMMHC forms a heterodimer with the key hematopoietic transcription factor RUNX1, and CBFbeta-SMMHC acts together with RUNX1 to activate the transcription of specific target genes (61). HDAC1 promotes transcriptional activation as a cofactor for the leukemic fusion protein CBFbeta-SMMHC (61). HDACs also act directly on proteins involved in tumor migration, metastasis and growth (97). For example, Api5 is a known anti-apoptotic and nuclear protein responsible for inhibiting cell death under conditions of serum starvation (97). The only known post-translational modification of Api5 is the acetylation of lysine 251 (K251) (97). p300 interacts with HDAC1 to regulate cell proliferation by regulating Api5 acetylation and stability (97). Inactivation of SIRT6 in cancer cells results in the accumulation of nuclear ACLY protein, increasing nuclear acetyl-CoA, which in turn drives site-specific histone acetylation and the expression of cancer cell adhesion and migration genes that promote tumor aggressiveness (98). Novel mechanism by which SIRT6 suppresses aggressive cancer cell phenotypes revealed and acetyl-CoA-responsive cell migration and adhesion genes identified as downstream targets of SIRT6 (98). Therefore, the regulatory mechanism of HDACs in tumors is difficult to be clearly described.

Class III HDACs are mainly located in mitochondria, which are the center of cellular energy metabolism (57). Acylated mitochondrial proteins are involved in many functions related to cellular metabolism, including TCA cycle, oxidative phosphorylation, nucleotide metabolism, amino acid metabolism, and urea cycle (99). SIRT can regulate energy production by regulating the acetylation and deacetylation of organisms involved in energy metabolism in mitochondria, thereby affecting cellular metabolism (7). For example, sirtuin 3 (Sirt3) is a key player in maintaining mitochondrial function and is involved in ATP production by regulating the acetyl and pyruvate dehydrogenase complex (PDH) (89). The underlying mechanism of SIRT is also related to the metabolic reprogramming of tumors (9). SIRT5 disruption-induced apoptosis is caused by a decrease in oxidative phosphorylation and glutamine utilization and an increase in mitochondrial superoxide, which is attenuated by ectopic superoxide dismutase 2 (93). SIRT5 controls and orchestrates key metabolic pathways in AML, so SIRT5 may be a potential therapeutic target in AML (93).

Class IV HDACs only contain HDAC11, which is highly expressed in HCC and is closely related to disease prognosis (96). Loss of HDAC11 promotes histone acetylation in the LKB1 promoter region, thereby activating the AMPK signaling pathway and inhibiting the glycolysis pathway, thereby increasing the transcription of LKB1, thereby inhibiting tumorigenesis and HCC progression (96). Histone deacetylases are abnormally expressed in clinical tumor patients and are associated with poor prognosis and survival (59). HDAC9 expression is positively associated with up-regulated genes in endocrine therapy-resistant breast cancer, and high HDAC9 levels are associated with poorer prognosis in patients treated with OHTam (82). HDAC10 regulates tumor stem cell properties in KRAS-driven lung adenocarcinoma, and HDAC10 regulates the stem-like properties of kras-expressing tumor cells by targeting SOX9 (85). The expression of SOX9 is significantly increased in HDAC10-depleted tumor cells, TGFβ pathway-related genes are enriched in HDAC10 knocked out tumor cells, and activation of TGFβ signaling contributes to the induction of SOX9 in HDAC10 knocked out lung adenocarcinoma cells (85). However, HDACs show activating activity in some tumors and inhibitory activity in some tumors, which suggests that their mechanism of action might not be a single one. SIRT1 may exert oncogenic effects by inactivating other tumor suppressors (eg, HIC1) and/or activating tumor-promoting genes (eg, *via* N-Myc stabilization or p53) or other proteins (cortatin) (100–102). There are interactions between HDACs. Studies have shown that inhibition or knockdown of HDAC1 and HDAC3 results in downregulation of HDAC7, which is associated with reduced histone 3 lysine 27 acetylation (H3K27ac) at transcription start sites (TSS) and super-enhancers (SEs), this is particularly evident in stem-like BrCa cells (80). Inactivation of HDAC7 can lead to suppression of the CSC phenotype, either directly or through the inhibition of HDAC1 and HDAC3, by downregulating multiple se-related oncogenes (80). HDAC7 may be a potential drug target (80).

HDACs inhibitors also have many adverse reactions in clinical application, such as drug resistance and toxic side effects (59). Aberrant expression of HDACs has also been shown to correlate with tumor resistance. HDAC8 increases the expression of p65, a key component of the NF-κB complex, and promotes the expression of IL-6 and IL-8 (103). This may be because HDAC8 can directly bind to the promoter of p65, increasing its transcription and expression. Thus, HDAC8 promotes DNR resistance in human AML cells by regulating IL-6 and IL-8 (103).

#### Acetyl coenzyme A

Acetyl Coenzyme A is a key precursor that used to synthesize acetyl. The progression of lysine acetylation can be controlled by regulating the concentration of acetyl-CoA. Acetyl-CoA is an important metabolite in cellular biological processes and is the only donor of acetyl groups during acetylation (104). Acetyl-CoA has different production pathways in different organelles. Acetyl-CoA produced in different organelles can be locally utilized in organelles, produced by decarboxylation of pyruvate in mitochondria, and produced by fatty acid β-oxidation in cytoplasm (105). ACLY, ACSS2, PDC can produce acetyl-CoA in organelles to regulate lysine acetylation (106). The interaction between lysine acetylation and acetyl-CoA is influenced by many factors, including the kinds of HATs, the acetyl-CoA/CoA ratio and intracellular pH gradient (107, 108).

Acetyl-CoA is derived from glycolysis and β-oxidation in the mitochondrial matrix, which ultimately leads to the production of cytoplasmic pyruvate,and enters the mitochondria for decarboxylation to form acetyl-CoA (109, 110). Branched-chain amino acids (i.e., valine, leucine, and isoleucine) can also be used to produce acetyl-CoA (111). Most of the acetyl-CoA in the cytoplasm comes from glutamine reductive carboxylation, which generates acetyl-CoA through the TCA cycle (112). Acetyl-CoA also has compartmentalized effects on protein acetylation. Acetyl-CoA exists in mitochondria, nucleus, and cytoplasm (105). Acetyl-CoA in mitochondria has a specific source pathway. Acetyl-CoA can pass through nuclear pores in the nucleus and cytoplasm. During the shuttle, acetyl-CoA has different abundances of acetyl-CoA in the nucleus and cytoplasm, and the occurrence of protein acetylation is also different (105). At the same time, studies have shown that the acetyl-CoA/CoA ratio may be a relevant regulator of HAT enzyme activity, rather than the absolute level of acetyl-CoA (105). This establishes a link between the nuclear and cytoplasmic abundance of acetyl-CoA and the epigenetic regulation of genes (105). In the process of tumorigenesis, abnormal expression of acetyl-CoA was also found. Acetyl-CoA can affect the proliferation, invasion and migration of tumor cells directly or by affecting protein acetylation (113). Acetyl-CoA induces cell growth and proliferation by promoting acetylation of histones at growth genes (113), and increase the levels of acetyl-CoA and acetylated histones to maintain the accelerated proliferation of cancer cells (105).

#### Reader

For histone acetylation to exert their biological functions, they also need to be combined with specific recognition proteins. Acetylated lysine in a protein will provide a reading site, recruit proteins with special structural domains, affect biological functions such as gene replication, gene transcription, and repair after DNA damage, and jointly participate in the regulation of gene expression (8). Recognition proteins can contain multiple different recognition domains that cooperate with PTM sites. Studies have shown that lysine-containing acetylation modification sites can be specifically recognized by proteins such as bromodomains, dual-PHD finger domains, and YEATS domains (8).

Four BET proteins have been identified in humans, BRD2, BRD3, BRD4 and the testis-specific protein BRDT (114). BRDT is only present in male germ cells (115). The BET family controls the transcription of various proinflammatory and immunoregulatory genes by recognizing acetylated histones (mainly H3 and H4) and recruiting transcription factors (such as RELA) and transcription elongation complexes (such as P-TEFb) to chromatin, thereby promoting the phosphorylation of RNA polymerase II and subsequent transcription initiation and elongation (116).

Localized in the nucleus, BRD2 can bind to hyperacetylated chromatin and play a role in transcriptional regulation through chromatin remodeling (115). BRD2 can regulate the transcription of the CCND1 gene and play a role in nucleosome assembly (117). Abnormal expression of BRD2 affects the development of various malignant tumors (118). For example, Runx3 forms a complex with BRD2 in a KRas-dependent manner in the early stages of the cell cycle, resulting in the inactivation of Runx3 and promoting the development of lung adenocarcinoma (118). Studies have shown that OCCC cells are susceptible to knockdown of epigenetic gene targets such as bromopseudomin and the extraterminal domain (BET) proteins BRD2 and BRD3, and targeting the BET proteins BRD2 and BRD3 in combination with PI3K-AKT inhibition may as a therapeutic strategy for ovarian clear cell carcinoma (119). The abnormal expression of BRD2 is also closely related to the drug resistance of patients. Studies have shown that BRD2 promotes drug resistance in adult T-LBL through the RasGRP1/Ras/ERK signaling pathway (120). Targeting BRD2 may be a new strategy to improve treatment efficacy and prolong survival in adults with T-LBL (120).

Localized to the nucleus, BRD3 is a chromatin reader that recognizes and binds hyperacetylated chromatin and plays a role in transcriptional regulation, possibly through chromatin remodeling and interactions with transcription factors (121). BRD3 regulates transcription by promoting the binding of the transcription factor GATA1 to its targets (122). The study found that BRD3 directly interacts with BCL6 and maintains the negative feedback regulatory loop of BCL6 (123). BRD2 and BRD3 preferentially associate with hyperacetylated chromatin throughout the length of transcribed genes *in vivo* (121). BRD2- and BRD3-associated chromatin was significantly enriched in H4K5, H4K12, and H3K14 acetylation reactions, and contained relatively less dimethylated H3K9 (121). Both BRD2 and BRD3 allow RNA polymerase II transcription by nucleosomes in a defined transcription system (121).

Localized in the nucleus, BRD4 is currently the most widely studied chromatin reader protein that recognizes and binds acetylated histones and plays a key role in the transmission of epigenetic memory across cell division and transcriptional regulation (124). Remains associated with acetylated chromatin throughout the cell cycle, and by preserving acetylated chromatin state and maintaining higher-order chromatin structure (125). Studies have shown that BRD4 is a transcriptional repressor of autophagy and lysosomal function (126). BRD4 plays a key role in regulating the transcription of signal-induced genes by binding to the P-TEFb complex and recruiting it to promoters. The P-TEFb complex is also recruited to the distal enhancer, an anti-pause enhancer that cooperates with JMJD6 (125). BRD4 and JMJD6 are required to form the transcriptionally active P-TEFb complex by replacing negative regulators such as HEXIM1 and the 7SK snRNA complex from P-TEFb, thereby converting it to the active form, which can then phosphorylate the C-terminal structure of RNA polymerase II Domain (CTD) (125). MYC regulates its own transcription by restricting its site for BRD4-mediated chromatin remodeling (127). The MYC-stabilizing kinase ERK1 regulates MYC levels directly or indirectly by inhibiting BRD4 kinase activity. These findings suggest that BRD4 negatively regulates MYC levels, which is counteracted by ERK1 activation (127).

BRD4 has three isoforms, BRD4 short isoform and BRD4 long isoform (128). There are two BRD4 short isoforms, which are spliced from other mRNAs. The short isoform of BRD4 promotes tumor metastasis, and the long isoform of BRD4 inhibits tumor metastasis and spread (128). Study shows BRD4 isoforms have opposing functions in breast cancer (128). The role of BRD4 in cancer is largely dependent on the long isoform (BRD4-L), and we demonstrated by isoform-specific knockdown and endogenous protein detection as well as transgene expression that the less abundant short isoform of BRD4 (BRD4-L) S) is oncogenic and BRD4-L has a tumor suppressor role in breast cancer cell proliferation and migration as well as breast tumor formation and metastasis (128). An isoform of BRD4 that acts as a chromatin insulator in DNA damage response pathways (129). Inhibits DNA damage response signaling by recruiting condensin-2 complexes to acetylated histones, leading to remodeling of chromatin structure, shielding this region from DNA by limiting the spread of histone H2AX/H2A.x phosphorylation injury response (129).

Due to the abnormal expression of BRD4 in various tumors, targeting BRD4 has emerged as a potential therapeutic strategy (130). For example, the expression of BRD4 in glioma was significantly higher than that in adjacent normal brain tissue (130). BRD4 inhibitors effectively penetrate the blood-brain barrier and target glioma tumor tissue, but have little effect on normal brain tissue (130). BRD4 is overexpressed in NFPA and GHPA, and the effects of BRD4 inhibitors on PA cells *in vitro* and *in vivo* were evaluated, so BRD4 is a promising therapeutic target for NFPA and GHPA (131).

BRD4 promotes the progression and metastasis of gastric cancer, and the abundance of BRD4 in human gastric cancer tissue is associated with shorter survival in patients with non-metastatic gastric cancer (132). BRD4 recognizes acetylated K146 and K187 on snails in an acetylation-dependent manner to prevent snails from recognition by their E3 ubiquitin ligases FBXL14 and β-Trcp1, thereby inhibiting snail polyubiquitination and proteases body degradation (132).

The mode of action of I-BET151 is due to the repression of transcription of key genes (BCL2, C-MYC and CDK6) by displacing BRD3/4, PAFc and SEC components from chromatin (133). This suggests that replacing BET proteins from chromatin is a potential epigenetic therapy for aggressive leukemia. BRDT (Bromodomain testis-specific protein), localized in the nucleus, exists only in male germ cells, and not often studied in tumors (115).

YEATS family proteins include YAF9, ENL, AF9, TAF14, SAS5 proteins (4). As the “readers” of protein acetylation, YEATS family proteins can combine with proteins to form various chromatin-related complexes with different complex functions, and play a role in chromatin remodeling and gene expression (4). YEATS family proteins are closely related to the occurrence of various malignant tumors. For example, ENL binds to acetylated histone H3, and co-localizes with H3K27ac and H3K9ac on the promoters of actively transcribed genes that are critical for leukemia (134). ENL is a regulator of leukemia. oncogenic transcriptional program (134), and an intact YEATS chromatin-reader domain was essential for ENL-dependent leukemic growth (135). YEATS4 overexpression enhances the malignant features of breast cancer cells, especially inducing epithelial-to-mesenchymal transition, and YEATS4 is associated with poor prognosis in breast cancer (136). YEATS protein promotes the proliferation of gastric cancer cells and affects tumor development by activating the Wnt/β-catenin signaling pathway (137). GAS41 is abundantly expressed in non-small cell lung cancer and is closely related to the proliferation of lung cancer cells (138). YEATS2, a target gene of HIF1α, promotes pancreatic cancer development under hypoxia (139).

Complex post-translational modifications are affected by many factors, one of which is the way the recognition site binds to the recognition protein. Initial studies believed that a post-translational modification recognition site can only bind to one recognition protein. The researchers found that a PTM recognition site can interact with multiple recognition proteins, eg. At the same time, a single recognition domain can also bind to multiple different protein PTMs, eg. Also, since recognition proteins include multiple distinct domains, synergy is extremely common in recognition proteins.

## Biological role of acetylation in cancer pathophysiology

**Figure 2:**
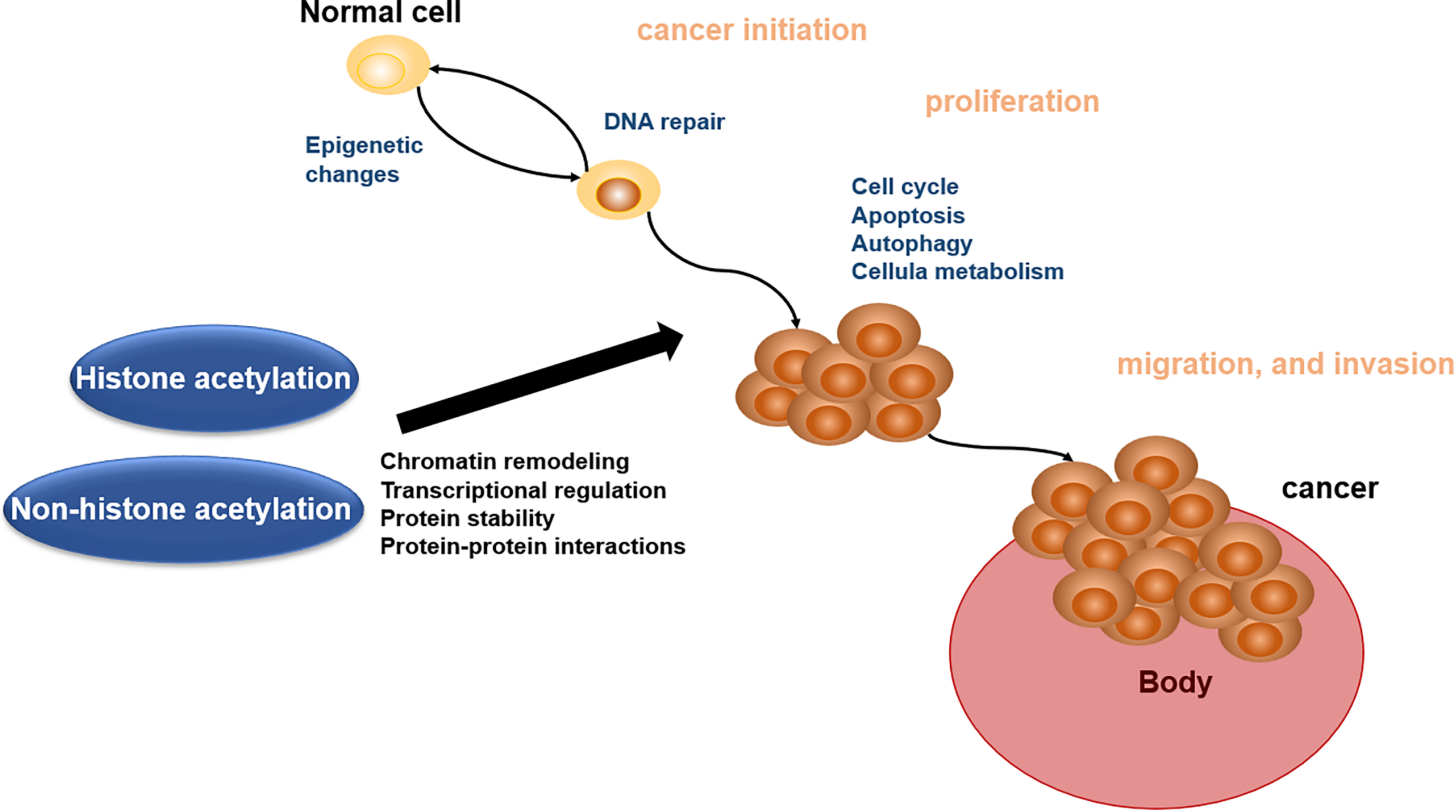
Biological role of histone acetylation and non-histone acetylation in cancer pathophysiology.

Acetylation of proteins is related to various kinds of cellular processes and human cancer (140). Here, we address the roles of acetylation in cancer cell apoptosis, autophagy, cell metabolism, cell cycle, proliferation, and migration and invasion, which will offer the basis for acetylation enzymes and BETs in reader as the important therapeutic targets (**Figure 2**).

### Role of acetylation in apoptosis

Apoptosis refers to the orderly death of cells controlled by genes, which is a normal programmed death in order to maintain the stability of the internal environment. In the process of cell apoptosis, it can be divided into the initiation stage, which receives apoptosis signals, interacts with apoptosis regulators, and then activates proteolytic enzymes, resulting in apoptosis (141). However, tumors have the characteristics of avoiding apoptosis, and abnormal apoptosis leads to abnormal tumor growth (142). The abnormal expressions of acetyltransferase and deacetylase can affect the normal apoptosis of cells. For example, PDCD5, a protein associated with apoptosis in human cells, binds to Tip60 and enhances the stability of Tip60 protein under stress-free conditions (143). PDCD5 increases Tip60-dependent K120 acetylation of p53 and is involved in p53-dependent expressions of apoptosis-related genes such as Bax (143). The combination of PDCD5 and Tip60 accelerates DNA damage-induced apoptosis, whereas knockdown of PDCD5 or Tip60 inhibits apoptosis-accelerating activity (143). HDAC1 and HDAC2 double knockout cells show significant activation of apoptosis (144). HDAC6 negatively regulates pro-apoptotic acetylation of p53 at K120 in mesenchymal stem cells (MSCs) (145). Studies have shown that targeting histone acetyltransferases and histone deacetylases can regulate tumor cell apoptosis, thereby affecting tumor growth and development (146). For example, histone deacetylase inhibitors induce apoptosis and autophagy in human neuroblastoma cells (147). Valproic acid induces cell cycle arrest and apoptosis *via* Hsp70 acetylation and inhibits proliferation of HER2-expressing breast cancer cells (148). When rRNA transcription was inhibited, nucleolar RNA content was reduced. The nucleolar protein Myb-binding protein 1A (MYBBP1A) translocates to the nucleoplasm and increases p53 acetylation as the level of nucleolar RNA content decreases (149). Acetylated p53 enhances p21 and BAX expression and induces apoptosis (149). Targeting protein acetylation to regulate tumor apoptotic activity can provide new therapeutic ideas for the clinical treatment of malignant tumors.

### Role of acetylation in autophagy

Autophagy is a special substance degradation pathway in cells, which depends on lysosomes for its action (10). The degradation substrates of autophagy include proteins and organelles. The probability of autophagy occurring in normal cells is low, and it mainly occurs in cells under abnormal conditions, such as starvation, hypoxia or organelle damage (150). There are three main types of autophagy. (i) The first type is microautophagy, in which lysosomes wrap a part of the cytoplasm into lysosomes and degrade them. (ii) The second is macroautophagy, which first generates an autophagosome (151). The double-membrane structure of the phagosome, the fruiting body contains the substances that need to be degraded in the cytoplasm, the autophagosome and the lysosome are combined to generate the autophagolysosome, and the acidic substances in the lysosome are used to degrade the autophagosome (151). Substances are degraded. (iii) The third is chaperone-mediated autophagy. Molecular chaperone-mediated autophagy uses heat shock protein 70 to bind to substrates with specific amino acid sequences and transport the substrates to lysosomes for further development (152). In 2004, Shao et al. found that HDAC inhibitor suberoylanilide hydroxamic acid β-D-glucur onide could induce autophagic death of cancer cells, and researchers gradually began to pay attention to the relationship between protein acetylation and autophagy (153). There is a close relationship between histone acetylation and cell autophagy. Histone acetylation can induce the occurrence of cell autophagy in the face of long-term stress, starvation and other harsh environments (154). The most widely studied is the relationship between H4K16ac and H3K56ac and autophagy. In eukaryotic cells, H4K16ac affects chromatin condensation and promotes gene transcription (155).

There is also a close link between non-histone acetylation and cell autophagy. Non-histone protein associated with autophagy that can be acetylated include transcription factors, autophagy-related proteins, and cytoskeletal proteins (7). The Fox O protein family is a transcriptional activator in eukaryotic cells, and acetylation can affect its biological activity. K on Fox O protein can be acetylated by HAT, and the activity of Fox O protein after acetylation is reduced, inhibiting DNA and its interaction, binding to regulate transcription (156). SIRT1 can also affect autophagy by regulating Fox O activity (157). TFEB protein is also a transcription factor that regulates the transcription of autophagy-related genes, such as LC3, and plays an important role in biological processes such as lysosomal biosynthesis and autophagy activation (158). The biological activity of the TFEB protein family is also affected by acetylation modification, and TFEB deacetylation can significantly enhance the autophagy and lysosomal function of cells (159). The TFEB-specific lysine acetylase is GCN5, which can acetylate the K276 and K279 sites of TFEB, affect the formation of TFEB dimers, interfere with the binding of TFEB and its targets, and inhibit autophagy happened (160). Acetylations affect subcellular localization, thereby affecting autophagy (161). In general, BmP300-mediated acetylation sequesters components of the BmAtg8-PE ubiquitin-like system in the nucleus, leading to inhibition of autophagy. Conversely, BmHDAC1-mediated deacetylation leads to nuclear-to-cytoplasmic transfer of components of the BmAtg8-PE ubiquitin-like system, promoting autophagy (161).

Protein acetylation is an important process regulating autophagy and plays an important role in the development of malignant diseases. The phosphorylation of ATG5 (T101) in the lesion tissue of glioblastoma patients is positively regulated by the acetylation modification of the hypoxia-induced autophagy regulator PAK1, which plays an important role in hypoxia-induced autophagy and promotes the occurrence and development of tumors (162). Targeting protein acetylation modification to regulate autophagy activity can provide new therapeutic ideas for clinical treatment of malignant tumors.

### Role of acetylation in cell metabolism

A major feature of tumors is uncontrolled proliferation, fueled by corresponding metabolic dysregulation (2). Tumors undergo metabolic reprogramming to promote tumor cell growth, division, invasion and migration. An abnormal response of tumor cell energy metabolism is called the Warburg effect (89). In the presence of oxygen, tumor cells reprogram glucose metabolism by limiting energy metabolism mainly to glycolysis, thereby generating energy for tumor growth (163). Lysine acetylation is a ubiquitous modification in enzymes that catalyze intermediate metabolism. Almost every enzyme in glycolysis, gluconeogenesis, tricarboxylic acid (TCA) cycle, urea cycle, fatty acid metabolism and glycogen metabolism is found to be acetylated in human liver tissue (10). All seven enzymes in the TCA cycle are acetylated (10). Acetylation occurs in most intermediate metabolic enzymes, and acetylation can directly affect the activity or stability of the enzyme (10). The bioenergetic preference of cancer cells promotes tumor acidosis, which in turn results in a marked reduction in glycolysis and glucose-derived acetyl-CoA (164). Protein acetylation affects tumor metabolism by affecting the TCA cycle. CBP acetylates STAT3 to undergo mitochondrial translocation, and STAT3 associates with pyruvate dehydrogenase complex E1, which in turn accelerates the conversion of pyruvate to acetyl-CoA, increases mitochondrial membrane potential and promotes ATP synthesis (165). SIRT5 removes the STAT3 acetyl group, thereby inhibiting its function in mitochondrial pyruvate metabolism (165). The protein also affects lipid metabolism in tumor cells and thus affects tumor development (166). Dynamic regulation of ME1 phosphorylation and acetylation affects lipid metabolism and colorectal tumorigenesis (166). The manner in which SIRT6 deacetylase antagonizes ACAT1 function involves mutually exclusive ME1 S336 phosphorylation and K337 acetylation (166). ACAT1 acetylates GNPAT at K128, which inhibits TRIM21-mediated GNPAT ubiquitination and degradation (167). GNPAT deacetylation by SIRT4 antagonizes the function of ACAT1. GNPAT inhibits TRIM21-mediated degradation of FASN and promotes lipid metabolism. promote the occurrence of liver cancer (167). Studies have shown that lysine acetylation controls metabolic activity by directly blocking the active site of the enzyme (168).

### Role of acetylation in cell cycle

Protein acetylation is closely related to gene transcription. Hyperacetylation promotes gene transcription and expression, while hypoacetylation inhibits gene transcription and expression (12). A large number of proteins involved in chromatin remodeling and cell cycle are acetylated (169). The cell cycle of tumor cells is greatly shortened and disordered. Studies have found that acetylation of tumor cells is also closely related to cell cycle progression (170). Protein acetylation affects tumor cell cycle progression by affecting chromatin remodeling, SIRT2 regulates H4K20me1 deposition through deacetylation of H4K16Ac (acetylation of H4K16), regulates chromatin localization, and affects cell cycle progression (169). Protein acetylation also has effects through the regulation of various factors in the cell cycle. For example, CDC2, a major cyclin-dependent kinase and regulator of S-phase progression and mitosis, is acetylated at residues K6 and K33 in CDC2 (25). SIRT1 interacts with CHK2 and is deacetylated at residure lysine 520, which inhibits CHK2 phosphorylation, dimerization, and thus activation (171). SIRT1 depletion induces CHK2 hyperactivation-mediated cell cycle arrest and subsequent cell death (171). Transcription factor Sp1 is a target of acetylation and is closely associated with cell cycle arrest in colon cancer cell lines (172). Simultaneous regulation of Api5 acetylation and deacetylation is an important factor in cell cycle progression (97).

### Role of acetylation in cell proliferation

Cancer cells have unlimited replicative potential with continuous proliferative signals (2). Normal cells and tissues release growth signals in an orderly manner, and these growth signals instruct cells to grow, divide and differentiate in an orderly manner, thereby ensuring the stability of cell numbers and the homeostasis of the internal environment, thereby maintaining normal tissue structure and function (2). However, tumor cell proliferation signals are abnormal and can continuously obtain proliferation signals from a variety of different pathways. In the abnormal proliferation of tumor cells, protein acetylation plays an important role. For example, acetylation at the K323 site of PGK1 is an important regulatory mechanism that promotes its enzymatic activity and cancer cell metabolism (173).

Acetyltransferase and deacetylase dynamically regulate the balance of acetylation, affecting the apoptosis and autophagy of tumor cells and other death methods, thereby affecting the proliferation of tumor cells. For example, Api5 is a known anti-apoptotic and nuclear protein responsible for inhibiting cell death under serum starvation conditions (97). The only known post-translational modification of Api5 is acetylation at K251. The K251 acetylation in Api5 is responsible for its stability, whereas the deacetylated form of Api5 is unstable (97). Inhibition of acetylation by p300 results in a decrease in Api5 levels, whereas inhibition of deacetylation by HDAC1 results in an increase in Api5 levels (97). Acetylation also affects the proliferation of tumor cells by affecting the activities of various metabolic enzymes in cells. For example, PKM2 K305 acetylation reduces PKM2 enzymatic activity and promotes its lysosome-dependent degradation through chaperone-mediated autophagy (CMA) (174). Degrade and promote tumor growth through chaperone-mediated autophagy (174). Ribonucleotide reductase (RNR) catalyzes the *de novo* synthesis of deoxyribonucleoside diphosphates (dNDPs), which provide dNTP precursors for DNA synthesis (175). Acetylation at residue K95 in RRM2 results in a reduction of the dNTP pool, DNA replication fork arrest, and inhibition of tumor cell growth *in vitro* and *in vivo* (175). P300 acetylates MAT IIα at K81 and destabilizes MAT IIα by promoting its ubiquitination and subsequent proteasomal degradation, inhibits tumor cell growth, and is reduced in human hepatocellular carcinoma (176). Inactivation of HDAC2 leads to elevated TPD52 acetylation, which impairs the interaction between TPD52 and HSPA8, resulting in impaired CMA function and tumor growth *in vivo* (177). Acetylation-dependent regulation of CMA oncogenic function in PCa by TPD52 suggests the possibility of targeting the TPD52-mediated CMA pathway to control PCa progression (177). p21 depletion converts KLF4 from a cell cycle inhibitor to a promoter of bladder cancer cell proliferation (178). Furthermore, KLF4 is acetylated in a p21-dependent manner to inhibit bladder cancer cell growth as a tumor suppressor (178). Since tumor cell proliferation is affected by acetylation modifications, drugs targeting acetylation can be used to treat abnormal tumor growth. For example, Rg3 extracted from ginsenosides has antiproliferative activity against melanoma by reducing HDAC3 and increasing p53 acetylation *in vitro* and *in vivo* (179). Therefore, Rg3 may serve as a potential therapeutic agent for the treatment of melanoma (179). Therapeutic modalities targeting acetyltransferases and deacetylases are also a potentially effective tumor treatment modality.

### Role of acetylation in migration and invasion

The development of tumor is divided into multiple stages. In the early stage, the primary lesion proliferates indefinitely, and after the formation of an obvious primary lesion, the function of the organ in which it is located is affected (2). Although the primary tumor is extremely malignant, the cause of death in most patients is the abnormal growth of metastatic tumors in sites other than the primary tumor (180). The reasons for these metastases are also unresolved and need to be discovered and solved urgently. Studies have found that protein acetylation is one of the important factors affecting tumor cell metastasis (6). For example, isocitrate dehydrogenase 1 (IDH1) is hyperacetylated in CRC primary tumors and liver metastases (181), sirtuin-2 is the deacetylase of IDH1, and SIRT2 overexpression significantly inhibits CRC cell proliferation, migration and invasion (181). COL6A1 is dysregulated in several human malignancies, and upregulation of H3K27 acetylation-activated COL6A1 promotes cell migration and invasion by inhibiting the STAT1 pathway in OS cells and promotes osteosarcoma lung metastasis (182). ZMYND8 acetylation of P300 at residues K1007 and K1034 is required for HIF activation and breast cancer progression and metastasis (183). TGF-β-activated kinase 1 (TAK1) stimulates phosphorylation by TGF-β and then induces acetylation of tubulin through αTAT1 activation, which subsequently activates AB cell migration and invasion (184). AFP acetylation promotes its oncogenic effects by blocking binding to the phosphatase PTEN and the pro-apoptotic protein caspase-3, thereby increasing signaling of proliferation, migration and invasion and reducing apoptosis (185). In HCC cells, hepatitis B virus X protein (HBx) and palmitic acid (PA) increased the levels of acetylated AFP by disrupting SIRT1-mediated deacetylation (185). AFP acetylation plays an important role in hepatocellular carcinoma progression (185). miR-15a-5p reduces histone H4 acetylation by inhibiting ACSS2 expression, inhibiting acetyl-CoA activity (186). miR-15a-5p inhibits lipid metabolism by inhibiting ACSS2-mediated acetyl-CoA activity and histone acetylation, thereby inhibiting a novel mechanism of lung cancer cell metastasis (186). In addition to histone acetylation affecting tumor cell invasion and migration, non-histone acetylation also affects tumor metastasis. For example, elevated levels of alpha-tubulin acetylation are sufficient reasons for the metastatic potential of breast cancer (187). Metastatic breast cancer cells exhibit high levels of alpha-tubulin acetylation, extending along microantenna (McTN) protrusions (187). Mutation of acetylation sites on α-tubulin and enzymatic regulation of this post-translational modification had a dramatic effect on McTN frequency and reattachment of suspended tumor cells (187). Reducing alpha-tubulin acetylation significantly inhibited migration but not proliferation (187). Targeting protein acetylation to affect tumor invasion and migration may serve as a potentially effective therapeutic strategy.

## Acetylation system-based targeted drugs in cancer

Research on abnormal protein acetylation in cancer mainly focuses on the mechanism of tumorigenesis, identification and prediction of new biomarkers for tumor invasion and migration, and tumor therapy. Since the process of protein acetylation is reversible, treating tumors can restore the acetylation process to normal levels for treatment. Therefore, some inhibitors of protein acetylation have also been approved for clinical treatment (59). For example, HAT inhibitors, HDAC inhibitors, HAT activators, and HDAC activators (59, 188, 189). HDAC activators are currently less studied.

Epigenetic regulation is an extremely promising strategy for the treatment of tumors, so many HAT- and HDAC-related modulatory drugs have been clinically tested (190). A research of NEO2734 in clinical trial revealed that there is an ongoing clinical trial. NEO2734 is a dual BET and CBP/p300 inhibitor targeting patients with advanced solid tumors and is in phase 1 clinical trials. Curcumin, a natural product-derived epigenetic modulator, the effect of curcumin on HDAC activity is variable and likely cell-line specific (190). Multiple clinical trials of curcumin have been completed, and other clinical trials are ongoing.

HDAC is considered to be a potential next-generation tumor therapy because HDAC inhibitors have been shown to have significant efficacy in a variety of tumor treatments (191, 192). Among them, vorinostat, romidepsin, panobinostat and belinostat have been approved by the US FDA for cancer treatment and are used in peripheral T-cell lymphoma, cutaneous T-cell lymphoma, and multiple myeloma (191, 192).

Vorinostat has been shown to be effective in the treatment of cutaneous T-cell lymphoma and is already in clinical use (192). Romidepsin regulates the expression of the immune checkpoint ligand PD-L1, and suppresses cellular immune function in colon cancer (193). Romidepsin has antitumor effects on several types of solid tumors (193). Romidepsin is used in clinical treatment of T-cell lymphoma (194). The safety and activity of panobinostat in relapsed/refractory Hodgkin lymphoma was also demonstrated in a multicenter phase II trial, and showed a significant reduction in tumor size (195). Belinostat has been found to be effective and well tolerated in patients with peripheral T-cell lymphoma (PTCL) or cutaneous T-cell lymphoma (CTCL) (196). Abexinostat is an extremely promising new HDAC inhibitor. Clinical trials have been carried out simultaneously in the United States and China. The main indications include hematological tumors (197, 198), metastatic sarcoma (199), breast cancer (200). There are also a number of drugs in clinical trials. Trichostatin A, for example, is in phase I clinical trials and is being tested in the clinic for tolerability in relapsed or refractory hematological malignancies. Ricolinostat is in phase II clinical trials for the treatment of multiple myeloma. The clinical development of HDAC inhibitors illustrates an extremely promising avenue for the treatment of tumors through epigenetic modulation.

### HAT inhibitors

HAT is one of the important targets of tumor therapy. HAT inhibitors are inhibitors of protein acetyltransferase, which can inhibit its activity and reduce the level of protein acetylation. Three types of HAT inhibitors have been reported, dual substrate inhibitors, natural compounds and synthetic compounds (201). HAT inhibitors are widely used in tumor treatment. Currently, the main researches are drug inhibitors targeting CBP/P300 and small molecule inhibitors of HAT domain (201) (**Table 3**).

**Table 3:** Classification and targets of HAT inhibitors in cancers.

Class	Drug	Targets	Cancer	references
dual substrate inhibitor	A-485	P300/CBP	Prostate cancer, Growth hormone pituitary adenoma, Human melanoma	(202–204)
	PU139	GCN5 P300 PCAF CBP	Neuroblastoma	(205)
	NEO2734	P300/CBP	Prostate cancer, Acute myeloid leukemia, Multiple myeloma	(206–208)
Natural compounds	Anacardic acid	P300 PCAF	Breast cancer	(209)
	Garcinol	PCAF	Colon cancer, Breast cancer, Prostate cancer, Head and neck cancer, Hepatocellular carcinoma	(210)
	Curcumin	P300/CBP		
	Delphinidin	P300/CBP	prostate cancer	(211)
synthetic compounds	C646	P300	Pancreatic cancer	(212)
	Acetaminophen	NAT2		
	WM-1119	KAT6A	Lymphoma	(213)
	Remodelin hydrobromide	NAT10		
	MG 149	Tip60	Colon cancer	(214)
	TH1834 dihydrochloride	Tip60	Breast cancer	(215)
	PF-9363	KAT6A/KAT6B		
	WM-3835	KAT7/MYST2	Osteosarcoma	(216)

Anacardiic acid, a natural compound extracted from natural plants, is a p300/CBP histone acetyltransferase inhibitor, significantly reduces the viability of PTEN-/- cells not in PTEN+/+ cells by inducing apoptosis (209). Delphinoside induces p53-mediated apoptosis in human prostate cancer LNCaP cells by inhibiting HDAC activity and activating p53 acetylation (211). Therefore, delphinidin may have a role in the prevention of prostate cancer (211). There are also synthetic compounds acting on HAT, targeting HAT as inhibitors to regulate intracellular acetylation homeostasis (210). A-485 competes with acetyl-CoA. A-485 selectively inhibits proliferation of lineage-specific tumor types, including several hematological malignancies and androgen receptor-positive prostate cancer (202). WM-3835 is a potent and highly specific HBO1 (KAT7 or MYST2) inhibitor that directly binds to the acetyl-CoA binding site of HBO1 33 WM-3835 activates apoptosis while inhibiting osteosarcoma (OS) cells proliferation, migration and invasion (216). WM-3835 has antitumor activity and potently inhibits the growth of osteosarcoma xenografts in mice (216). TH1834 dihydrochloride is a specific Tip60 (KAT5) histone acetyltransferase inhibitor (215). TH1834 dihydrochloride induces apoptosis and increases DNA damage in breast cancer cells. TH1834 dihydrochloride does not affect the activity of the related histone acetyltransferase MOF. Anticancer activity (215). Combination therapy of CK1 inhibitor SR3029 and Tip60 inhibitor MG149 had stronger inhibitory effects on β-catenin acetylation, transcription of Wnt target genes, and viability and proliferation of colon cancer cells (214). Transcriptional activity of β-catenin can be regulated through the CK1δ/ϵ-β-catenin-Tip60 axis, which may be a potential therapeutic target for colon cancer (214).

### HAT activators

HAT activators are activators that act on protein acetyltransferases and can activate acetyltransferases to increase the level of protein acetylation. For example, CTB can induce acetylation of P53 protein by increasing the expression of P300, thereby inducing significant cell death in MCF-7, but it may be well tolerated in MRC-5 (217). Therefore, CTB can be applied in cancer treatment (217). The research on HAT activators is not very extensive, and most of them are activators targeting the CBP/P300 complex (217) (**Table 4**).

**Table 4:** Targets of HAT activators and associated cancers.

Drug	Targets	Cancer	References
CTB	P300	Breast cancer	(217)
TTK21	CBP/P300		
CTPB	P300		
I-CBP112	CBP/p300	Leukemia	(218)
YF-2	CBP PCAF GCN5		

### HDAC inhibitor

HDACs are found to be abnormally expressed in malignant tumors (219). The expression of HDACs is closely related to clinical treatment prognosis and tumor occurrence and development. In liver cancer, inhibition of HDAC2 expression can promote histone acetylation in the promoter region of MIR22HG, thereby upregulating the expression of MIR22HG, promoting the production of miR-22-5p, and ultimately increasing the sensitivity to radiotherapy (64). In acute B lymphocytic leukemia, inhibits the activity of HDAC3, which enhances the sensitivity of acute B lymphocytic leukemia cells to drugs by inhibiting the JAK/signal transducer and activator of transcription 3 signaling pathway (220). Inhibition of HDAC8 activity causes cytotoxic effects, cell cycle arrest in human monocytic leukemia followed by apoptosis, and cytostatic effects in p53-deficient human myelocytic leukemia cells (73). SIRT1/2 inhibition results in HSPA5 acetylation and dissociation from EIF2AK3, leading to endoplasmic reticulum stress response, which in turn upregulates ATF4 and dit4, triggering autophagy (86). Sirtuins have become a promising target for a novel class of anti-cancer drugs. HDAC inhibitor can reverse this phenomenon and reactivate the expression of tumor suppressors, and HDAC inhibitor can act on histone acetylation and non-histone acetylation to inhibit tumor growth, invasion and metastasis, and has become a clinically effective anti-tumor drug (221) (**Table 5**).

**Table 5:** Classification and targets of sirtuins in cancers.

Class	Drug	Targets	Cancer	Reference
Hydroxamates	Vorinostat	HDACs 1, 2, 3, 6	CTCL, BCR-ABL-negative myeloproliferative neoplasms, Triple-negative breast cancer, Melanoma	(222–225)
	Panobinostat	HDACs	Multiple myeloma, Prostate cancer, Acute myelogenous leukemia	(226, 227)
	Trichostatin A (TSA)	HDACs 7, 8	Esophageal squamous, Cholangiocarcinoma, Cholangiocarcinoma, Osteosarcoma	(228–231)
	Belinostat	HDACs	PTCL, Pancreatic cancer, Lung squamous cell carcinoma, Breast cancer	(232–235)
	Dacinostat (LAQ824)		Medulloblastoma, Malignant Melanoma	(236, 237)
	Givinostat	HDACs	Chronic myeloproliferative neoplasms, Hematological malignancies	(238, 239)
	Resminostat	HDACs	Hodgkin’s lymphoma, Hepatocellular carcinoma, Lymphoma	(240, 241)
	Abexinostat	HDAC 1	Lymphoma, Leukemia, Lymphocytic	(198)
	Quisinostat	HDACs	Lymphoma, Neoplasms, Myelodysplastic syndromes, Hepatocellular carcinoma, Neuroblastoma, Tongue squamous cell carcinoma	(242–244)
	CUDC-101	HDACs	Lymphoma, Pancreatic cancer, Liver cancer, Breast cancer, Gastric cancer	(245, 246)
	CUDC-907	HDACs	Lymphoma, Solid tumors, Breast cancer, Multiple myeloma, NUT midline carcinoma	(247, 248)
	MPT0E028	HDACS 1, 2, 6		
	CHR-3996	HDACs		
	LMK235	HDACs 4, 5		
Short-chain fatty acids	Valproic acid (VPA)	HDACs 2, 9	Acute myeloid leukemia Cholangiocarcinoma	(229, 249)
	Phenylbutyrate	HDACs 1-11	Oral squamous cell carcinoma	(250, 251)
	Pivanex (AN-9)	HDACs	Lung cancer, Liver cancer	(252)
	AR-42	HDACs	Acoustic neuroma, Testicular lymphoma, Intraocular lymphoma, Esophageal squamous cell carcinoma, Adult T-cell leukemia, Lymphoma osteolytic bone tumors, Vestibular schwannoma	(253, 254)
Cyclic tetrapeptide	Romidepsin (Depsipeptide/FK228)	HDACs 1, 2, 4, 6	CTCL	(255)
Benzamides	Mocetinostat (MGCD0103)	HDACs 1, 2, 3	Lymphoma, Urothelial carcinoma, Relapsed and refractory, Myelodysplastic syndrome, Metastatic leiomyosarcoma	(256)
	Entinostat (MS-275)	HDACs	Breast cancer, NCSLC, Osteosarcoma, Ovarian cancer, Hematologic malignancies, Oral squamous cell carcinoma	(257–262)
	Tacedinaline (CI-994)		Lung cancer, Multiple myeloma	
	Chidamide	HDAC 1, 2, 3, 10	T-cell lymphoma	(263)
	Ricolinostat (ACY-1215)	HDAC 6	Multiple myeloma	(264)

Studies have shown that HDAC inhibitor has a significant inhibitory effect on P53, HSP90, NF-κB factors and multiple dephosphorylation enzymes, and a variety of HDAC inhibitors have been developed (59). The FDA has developed and approved several HDAC inhibitors for clinical cancer treatment. HDAC inhibitors are mainly divided into five categories according to different structures, including short-chain fatty acids, amides, hydroxamic acids, cyclic peptides, and chemical substances extracted from plants (265). Among histone deacetylase inhibitors, fatty acids are one of the less commonly used inhibitors. Valproic acid is an anticonvulsant drug that has been used clinically in bipolar disorder (266). The study found that valproic acid can also inhibit histone deacetylase 9, affect Notch cell signaling, and inhibit the activity of human neuroblastoma cells (267). The HDAC inhibitor of the benzamide class is the first inhibitor that selectively targets class I HDACs. There are also a large number of benzamide drugs in clinical trials for tumor treatment (59). The enzyme kinetics study of aminobenzamide-based HDAC inhibitors shows that the aminobenzamide motif has a tight binding mechanism (slow start/slow shutdown) unlike the classical fast-on/fast-off kinetics of hydroxamic acid-based HDAC inhibitors (268).

Hydroxamic acid HDAC inhibitors are the first class of HDAC inhibitors to be developed (59). Vorinostat is the first HDAC inhibitor on the market. At appropriate concentrations, vorinostat can inhibit HDAC1, 2, 3, 6, inhibit the activity of HDAC, and lead to significant hyperacetylation of H4 at residues lysine 5, 8, 12, 1, and 6 (269). These hyperacetylation are closely related to transcriptional changes, and vorinistat can simultaneously increase or decrease the transcription of specific genes in tumor cells, suggesting that HDAC inhibitor can have completely opposite effects throughout the genome (265). Virinostat is currently approved for the treatment of cutaneous T-cell lymphoma (CTCL). Studies have shown that vorinostat has activity in the treatment of recurrent glioblastoma multiforme (270). Clinically, it can be used in combination with other drugs to treat tumors (270). Vorinostat is clinically used in combination with gefitinib in the treatment of lung cancer to enhance the induction of apoptosis of lung cancer cells (271). Panobinostat is involved in many biological processes, including DNA replication and repair, chromatin remodeling, gene transcription, cell cycle progression, protein degradation and cytoskeleton reorganization (226). For example, in prostate cancer, Panobinostat reverses HepaCAM gene expression and inhibits proliferation by increasing histone acetylation (226). Panobinostat can also be used in combination with other drugs to improve treatment efficiency, such as in acute myeloid leukemia, studies have shown that the combination of panobinostat differentiation and arsenic trioxide apoptosis can significantly improve survival (272). Another HDAC inhibitor is SIRT inhibitor, inhibition of SIRT1 and SIRT2 induces cancer cell apoptosis and plays multiple roles in regulating autophagy (86). Salermide in NSCLC cells, inhibiting SIRT1 and 2 by acetylating HSPA5, and then activating ATF4 and dit4 to inhibit the mTOR signaling pathway, thereby inducing pro-survival autophagy (86). Ginsenoside Rg1 inhibits cell proliferation and induces cellular senescence in acute myeloid leukemia cells CD34+CD38- leukemia stem cells by activating Sirtuin 1 (SIRT1)/tuberous sclerosis complex 2 (TSC2) signaling pathway (273). Capsaicin attenuates cell migration by enhancing corticosteroid and -catenin acetylation in bladder cancer cells through SIRT1 targeting and inhibition (274). Capsaicin-reduced cell migration is associated with downregulation of sirtuin 1 (SIRT1) deacetylase, possibly through proteasome-mediated protein degradation (274). Combination therapy of SIRT1/2 inhibitor and drug autophagy inhibitor is an effective therapeutic strategy (86). Some studies have found that synthetic HDAC inhibitors may have toxic side effects such as atrial fibrillation, researchers turned their attention to natural inhibitors extracted from plants (59). Plant-derived inhibitors also showed good activity in inhibiting tumors. For example, hawthorn polyphenol extract (HPE) can significantly reduce ROS levels, apoptosis and inflammation-related factor expression in cells, and also inhibit AMPK/SIRT1/NF-κB and miR-34a/SIRT1/p53 pathways by regulating acetylation (275). Pathway is involved in hyperglycemia-induced inflammation and apoptosis of human retinal epithelial cells (275). These inhibitors can significantly inhibit tumor proliferation, migration and invasion, and can induce apoptosis and induce autophagy (59). However, the application of these inhibitor drugs in clinical practice requires more in-depth research.

### BET inhibitor

As a scaffold protein, BET can read epigenetic code, recognize histone acetylation or non-histone acetylation, and regulate gene expression, and play an important role in cell function (115). However, abnormal expression of BET leads to abnormal gene expression, resulting in abnormal cell function, which is related to the development of many malignant diseases. The study found that the abnormal expression of BRD4 is related to glioma, and the expression in glioma is significantly higher than that in normal tissue (130); BRD4 inhibitors effectively penetrated the blood-brain barrier and targeted glioma tumor tissue, but had little effect on normal brain tissue (130). Therefore, BRD4 is a target for the treatment of glioma (130). Targeting BET protein therapy is a very promising tumor treatment strategy. The BET-bromodomain-specific inhibitors JQ1, I-BET and I-BET151 represent initial successes in the development of BET inhibitors (276). The small molecule BET inhibitor drug, JQ1, is a potent growth inhibitor for many cancers and holds promise for cancer therapy (276). However, studies have found that JQ1 can activate other oncogenic pathways and may affect epithelial-to-mesenchymal transition (EMT) (276). That is to say, JQ1 has an unexpected role in promoting prostate cancer invasion (276). In the application of tumor treatment, attention should be paid to the possible toxic and side effects of JQ1. BET inhibitor treatment in HCC cell lines reduces cell migration by downregulating SMARCA4 (277). GS-5829 inhibits CLL cell proliferation and induces leukemia cell apoptosis by deregulating key signaling pathways such as BLK, AKT, ERK1/2, and MYC (278). BRD2 supports borderline activity and raises the possibility that pharmacological BET inhibitors may partially affect gene expression by interfering with regional borderline function (279). Disruption of negative autoregulation by BET inhibitor (BETi) leads to a marked increase in BCL6 transcription, which further activates the mTOR signaling pathway by inhibiting tumor suppressor death-associated protein kinase 2 (123).

The effectiveness of BET-specific targeted inhibitors is often affected by tumor drug resistance (280). There is also an urgent need to address the issue of BET inhibitor resistance. Prostate cancer-associated SPOP mutations confer resistance to BET inhibitors by stabilizing BRD4 (281). Tumor-suppressive effects of SPOP in prostate cancer, where it acts as a negative regulator of BET protein stability, and also provides a molecular mechanism for resistance to BET inhibitors in individuals with prostate cancer carrying SPOP mutations (281). Prostate cancer-associated SPOP mutants display impaired binding to BET proteins, leading to reduced proteasomal degradation and accumulation of the protein in prostate cancer cell lines and patient specimens, and causing resistance to BET inhibitors (282). Transcriptomic and BRD4 enzymatic analysis revealed enhanced expression of GTPase RAC1 and cholesterol biosynthesis-related genes, and activation of AKT-mTORC1 signaling due to BRD4 stabilization (282). Resistance to BET inhibitors in SPOP-mutant prostate cancer can be overcome by combination with AKT inhibitors and further supports the evaluation of SPOP mutations as biomarkers to guide BET inhibitor-directed therapy in prostate cancer patients (282).

Although research on BET inhibitors is still a research focus, the combination use of BET inhibitors with other drugs is also being explored. BET inhibitors can be used in combination with other types of inhibitors in order to promote the therapeutic effect or reduce adverse reactions (283). For example, the combination of BET inhibitor I-BET762 and PARP inhibitor Talazoparib Synergy is used in the treatment of SCLC and has a synergistic effect (283). At the same time, a strategy of combined application of HDAC inhibitor and JQ1 inhibitor has shown good efficacy in the treatment of AML (284). Based on the combination drug strategy, dual-target inhibitors of HDAC and BET are also being developed, and have shown more significant efficacy than single-target inhibitors in the treatment of pancreatic cancer (285). This multi-targeted drug can ensure the efficacy and durability of the anti-cancer effect, and this combination approach also reduces the possibility of tumor resistance (285). This provides a new scope of research for BET inhibitors in the treatment of tumors. BET and HDAC inhibitors are synergistic at reduced doses, suggesting a potential approach to avoid overlapping toxicities of the two drug classes (280). The combination of CPI-0610 with a PRAP inhibitor has been found to better address PRAP inhibitor resistance in ovarian cancer patients (286). It also proposes new therapeutic strategies to address PARP inhibitor resistance using drugs already approved or in clinical development that have the potential to rapidly transform and benefit a broad range of ovarian cancer patients (286) (**Table 6**).

**Table 6:** Targets of BET inhibitors and related cancers.

Name	Targets	Cancer	References
Molibresib	BRD2, BRD3, BRD4	Hematological malignancies	(287)
ARV-771			
HJB97			
Birabresib	BRD2, BRD3, BRD4	Solid tumor	(288)
MS436	BRD4		
BRD4 D1-IN-2	BRD4		
AGB1			
JQ1		Prostate cancer, Retinoblastoma, Ovarian cancer	(276, 289, 290)
I-BET762		SCLC, Pancreatic ductal adenocarcinoma, Hepatocellular carcinoma	(283, 291, 292)
I-BET151	BRD4	Ovarian cancer, Multiple myeloma, MLL-fusion leukemia,	(133, 293, 294)
CPI-0610		Multiple myeloma, Ovarian cancer	(286, 295)
PFI-1		Prostate cancer	(296)
I-BET726		Human skin squamous cell carcinoma, Neuroblastoma	(297, 298)
ABBV-744		Acute myeloid leukemia, Prostate cancer	(299, 300)

## Future perspectives

Tumor is currently the most troublesome problem in human life and seriously affects human health. The development of tumors is affected by many factors, including genetic factors and epigenetic factors (6). The development of tumor is the result of the joint influence of many factors (6). Protein acetylation is at the junction of genetics, epigenetics and tumor microenvironment (9). Protein acetylation is affected by many aspects to promote the occurrence and development of tumors (9). For example, protein acetylation writer, eraser, and reader may be abnormally expressed (7). Regulatory factor or regulatory factors aberrantly promote tumorigenesis and are associated with multiple malignant phenotypes of tumors. The study of protein acetylation provides a deeper understanding of tumor-related mechanisms, facilitates the discovery of potentially effective biomarkers and therapeutic targets, and facilitates the discovery and application of therapeutic drugs (11). At the same time, it is beneficial to solve the drug resistance and recurrence of tumors. At the same time, we also emphasize the strengthening of these studies on protein acetylation in different cancers, combined with PPPM in clinical practice for the treatment of malignant tumors (301).

## Conclusions

This review summarized current studies about the role of protein acetylation in tumors and related targeted therapy drugs, including the classification of protein acetylation, related regulators of protein acetylation, the pathological role of protein acetylation in tumors, and targeted proteins acetylated drugs. Protein acetylation affects various physiological functions of tumors and is therefore associated with tumor development and progression. Protein acetylation plays an important role in the link between cancer pathology and post-translational modifications. Therefore, protein acetylation plays an important role in tumor therapy. Drugs about protein acetylation have been extensively studied. Drugs targeting protein acetylation have promising applications in tumor therapy, and combined use with other pathway drugs is a potential therapeutic strategy.

## Author contributions

JY collected and analyzed literature, and wrote the manuscript. CS participated in partial literature analysis. XZ conceived the concept, designed the manuscript, coordinated and critically revised manuscript, and was responsible for its financial supports and the corresponding works. All authors contributed to the article and approved the submitted version.

## Acknowledgments

The authors acknowledge the financial supports from the Shandong First Medical University Talent Introduction Funds (to XZ), Shandong First Medical University High-level Scientific Research Achievement Cultivation Funding Program (to XZ), the Shandong Provincial Natural Science Foundation (ZR202103020356/ZR2021MH156 to XZ), and the Academic Promotion Program of Shandong First Medical University (2019ZL002).

## Conflict of interest

The authors declare that the research was conducted in the absence of any commercial or financial relationships that could be construed as a potential conflict of interest.

## Publisher’s note

All claims expressed in this article are solely those of the authors and do not necessarily represent those of their affiliated organizations, or those of the publisher, the editors and the reviewers. Any product that may be evaluated in this article, or claim that may be made by its manufacturer, is not guaranteed or endorsed by the publisher.

## Glossary

**Table:**

ACLY	ATP- citrate lyase
ACSS2	Acyl- CoA synthetase short- chain family member 2
Api5	Apoptosis inhibitor 5
ATAT-1	Alpha-tubulin N-acetyltransferase 1
BET	Bromodomain and extra-terminal
BETi	Bromodomain and extra-terminal inhibitor
BRD2	Bromodomain 2
BRD3	Bromodomain 3
BRD4	Bromodomain 4
BRDT	Bromodomain testis-specific protein
CBFB	Core-binding factor beta
CBP	CREB- binding protein
CMA	Chaperone-mediated autophagy
CTCL	Cutaneous T-cell lymphoma
CTD	C-terminal structure of RNA polymerase II Domain
EMT	Epithelial-to-mesenchymal transition
ERalpha	Estrogen receptor alpha
ESCO1	Establishment of sister chromatid cohesion N-acetyltransferase 1
ESCO2	Establishment of sister chromatid cohesion N-acetyltransferase 2
Fox O	Forkhead box-containing protein
O subfamily	
GCN5	General control of amino acid synthesis protein 5
H3	Histone 3
H3K14	Histone H3 lysine 14
H3K27	Histone H3 lysine 27
H3K27ac	Histone H3 lysine 27 acetylation
H3K56ac	Histone H3 lysine 56 acetylation
H3K9	Histone H3 lysine 9
H3K9ac	Histone H3 lysine 9 acetylation
H4	Histone 4
H4K12	Histone H4 lysine 12
H4K16ac	Histone H4 lysine 16 acetylation
H4K20me1	Histone H4 lysine 20 mono-methylation
H4K5	Histone H4 lysine 5
HAT	Histone acetyltransferase
HAT1	Histone acetyltransferase 1
HBO1	Histone acetyltransferase binding to ORC1
HBx	Hepatitis B virus X protein
HCC	Hepatocellular carcinoma
HDAC	Histone deacetylase
hnRNPA1	Heterogeneous nuclear ribonucleoprotein A1
HPE	Hawthorn polyphenol extract
Hsp70	Heat shock protein 70
HSPA5	Heat shock protein family A (Hsp70) member 5
HSPA8	Heat shock protein family A (Hsp70) member 8
IDH1	Isocitrate dehydrogenase 1
IL-6	Interleukin- 6
IL-8	Interleukin- 8
McTN	Microantenna
MOF	Males absent on the first
MOZ	Monocytic leukemia zinc finger protein
MSCs	Mesenchymal stem cells
mTOR	Mechanistic target of rapamycin kinase
Naa10	N-alpha-acetyltransferase 10
NAT1	Arylamine N-acetyltransferase 1
NF-PitNETs	Nonfunctional pituitary neuroendocrine tumors
NF-kB	Nuclear factor k-B
NSCLC	Non-small cell lung cancer
OCCC	Ovarian clear cell carcinoma
OS	Osteosarcoma
PA	Palmitic acid
PDH	Pyruvate dehydrogenase complex
PHD	Plant homeodomain-linked
PKM	Pyruvate kinase
PKM2	Pyruvate kinase M1/2
PRAP	Proline-rich acidic protein
PTM	Post-translational modification
RNR	Ribonucleotide reductase
SCLC	Small Cell Lung Cancer
Ses	Super-enhancers
SIRT1	Sirtuin 1
SIRT2	Sirtuin 2
SIRT3	Sirtuin 3
SIRT4	Sirtuin 4
SIRT5	Sirtuin 5
SIRT6	Sirtuin 6
snRNA	Small nuclearRNA
TAK1	TGF-β-activated kinase 1
TAT1	α-tubulin N- acetyltransferase 1
TCA	Tricarboxylic acid
Tip60	60 kDa Tat- interactive protein
TSC2	Tuberous sclerosis complex 2
TSC2	Tuberous sclerosis complex 2
TSS	Transcription start sites
YEATS2	YEATS domain containing 2
YEATS4	YEATS domain containing 4
YopJ	Serine/threonine-protein acetyltransferase YopJ.
